# Supplementary material for: A Cross-Tissue Investigation of Molecular Targets and Physiological Functions of Nsun6 Using Knockout Mice
Source: Int J Mol Sci. 2022 Jun 13;23(12):6584. doi: 10.3390/ijms23126584 (PMC9224068; doi:10.3390/ijms23126584)
Supplement: Supplementary file 1 [file ijms-23-06584-s001.zip › supplementrary figure legends.pdf]

Figure legends of supplementary figures

Figure S1. Confirmation of Nsun6 KO at protein level. Liver, spleen, and heart tissues were used to confirm the KO of Nsun6 using Western blot.

Figure S2. The features of NSUN6-dependent m5C sites. A. IGV plots presenting a Nsun6-dependent m5C site on gene Igfbp6 in WT and KO of small intestine. The sequence represents the genome sequence, and the red bars represent the C-T mismatches induced by bisulfite conversion. The site indicated by the arrow is the m5C site, where a proportion of C was converted to T in WT tissue. B. Nucleotide frequency of 80 Nsun6-dependent m5C sites and their 10 nt flanking regions. C. IGV plots presenting m5C modification rates of Furin and Nectin2, whose orthologous genes in human are highly modified. D. Protein levels of Nsun6 in different mouse tissue detected by Western blot. E. GO enrichment analysis of all m5C sites in heart.

Figure S3. The analysis of Nsun6-KO across tissues. A. The volcano plots presenting the DEGs identified in heart, small intestine, and kidney, respectively. B. GO analysis of DEGs in Nsun6-KO liver.

Figure. S4 Lymphocytes development was intact in spleen of Nsun6 KO mice. A. Flow cytometric analysis of ratio of B cells ($B220^{+}$), macrophages ($B220^{-}F4/80^{+}$), Gr-1+ cells ($Gr\text{-}1^{+}$), natural killer cells ($B220^{-}NK1.1^{+}$), CD4+ T cells ($CD4^{+}CD8^{-}$), CD8+ T cells ($CD4^{-}CD8^{+}$), and CD4+ CD8+ T cells ($CD4^{+}CD8^{+}$). B. The statistical graph of B cells ($B220^{+}$), macrophages ($B220^{-}F4/80^{+}$), Gr-1+ cells ($Gr\text{-}1^{+}$), natural killer cells ($B220^{-}NK1.1^{+}$), CD4+ T cells ($CD4^{+}CD8^{-}$), CD8+ T cells ($CD4^{-}CD8^{+}$), and CD4+ CD8+ T cells ($CD4^{+}CD8^{+}$).
