# Supplementary figures and images for: A Cross-Tissue Investigation of Molecular Targets and Physiological Functions of Nsun6 Using Knockout Mice
Source: Int J Mol Sci. 2022 Jun 13;23(12):6584. doi: 10.3390/ijms23126584 (PMC9224068; doi:10.3390/ijms23126584)

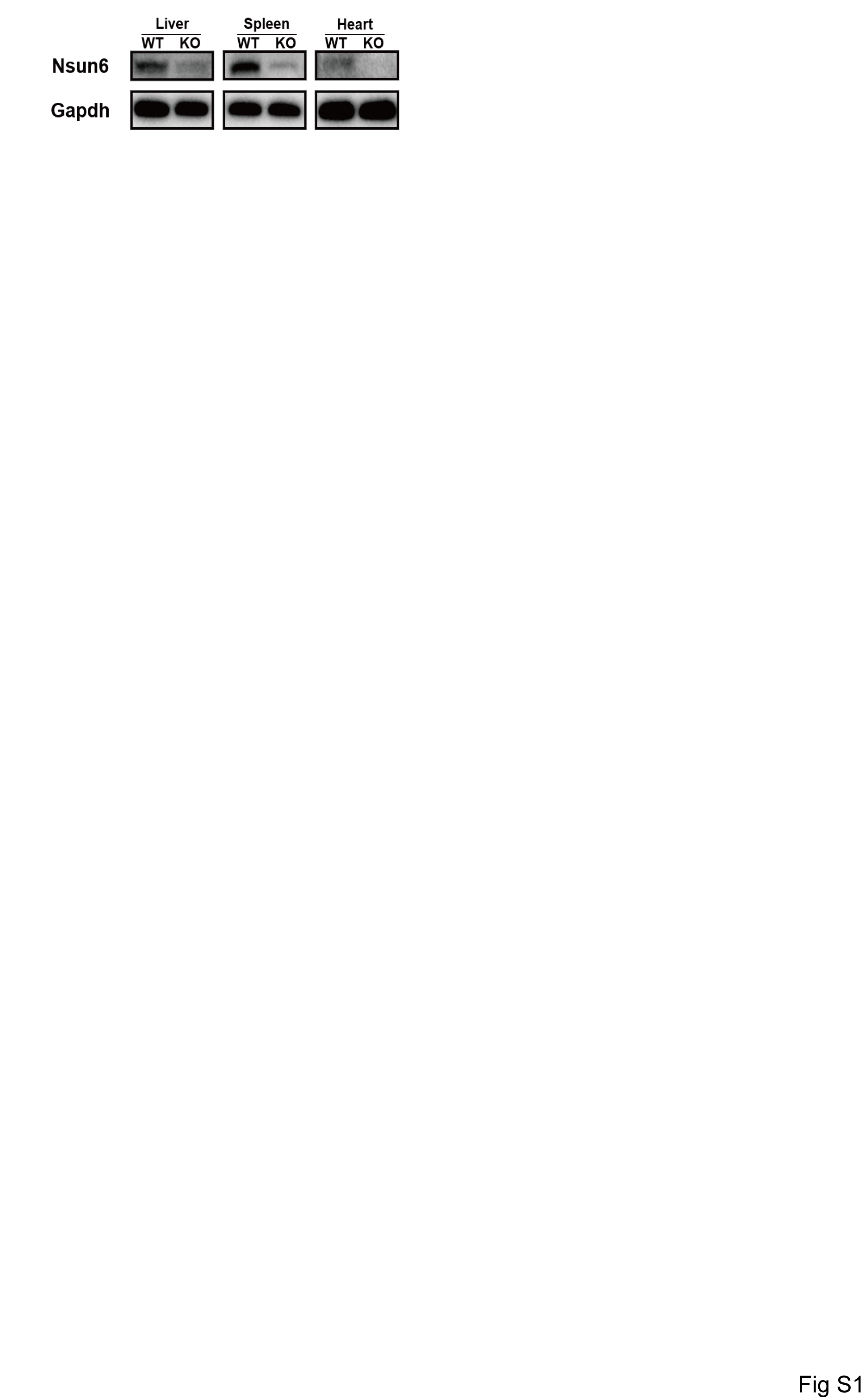

Supplement: Supplementary file 1 [file ijms-23-06584-s001.zip › Supplementary fig S1.tif]

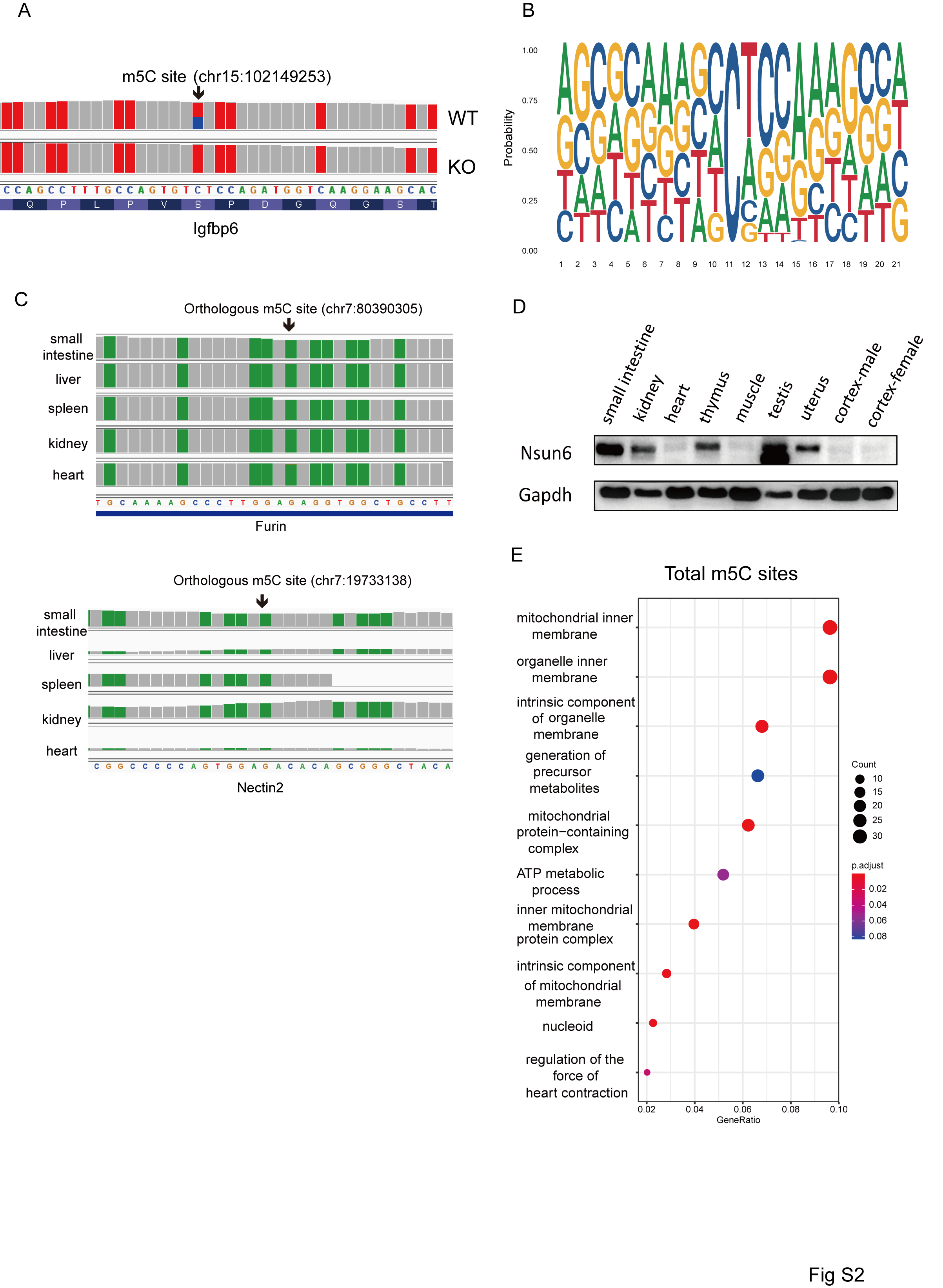

Supplement: Supplementary file 1 [file ijms-23-06584-s001.zip › Supplementary fig S2.tif]

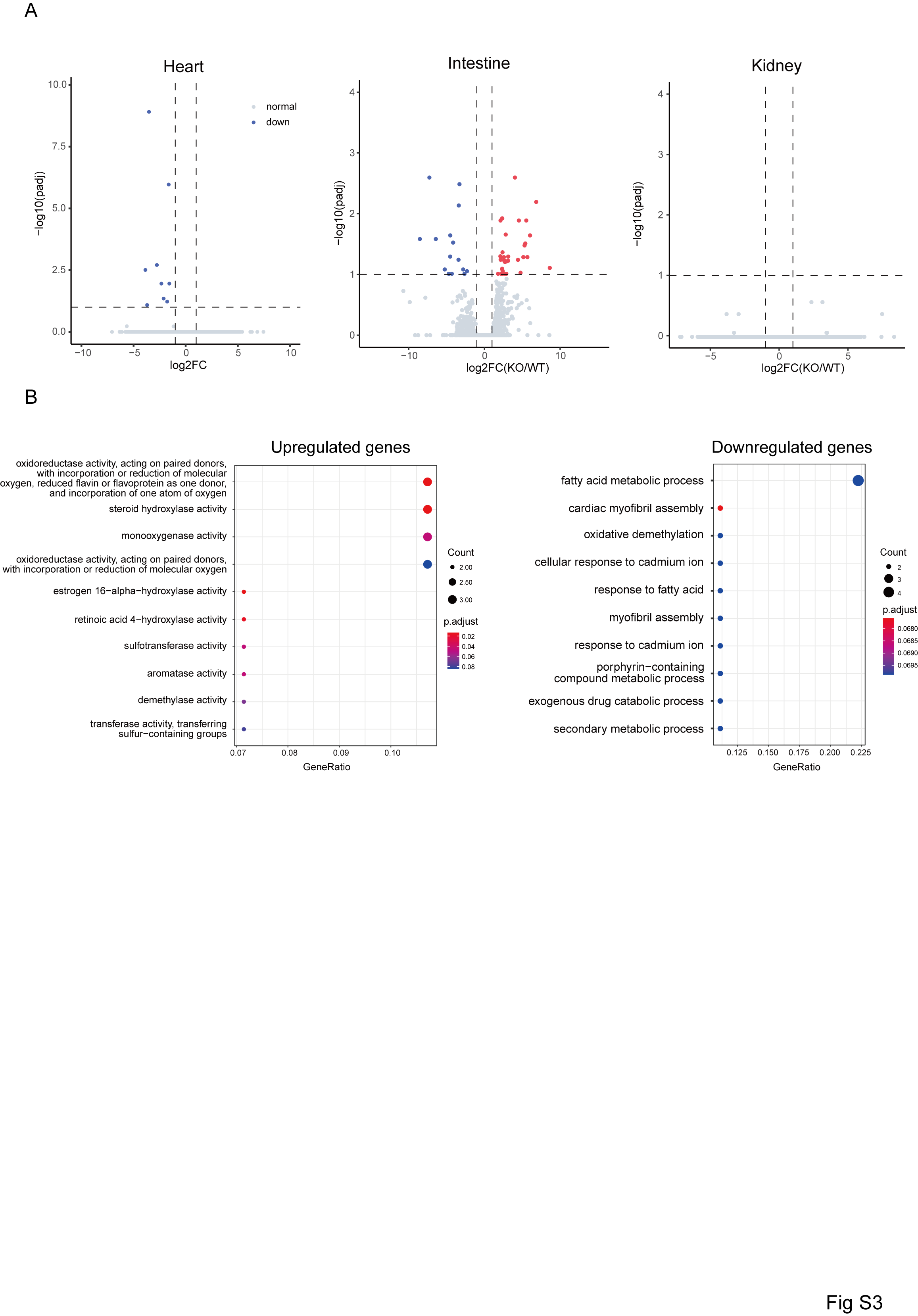

Supplement: Supplementary file 1 [file ijms-23-06584-s001.zip › Supplementary fig S3.tif]

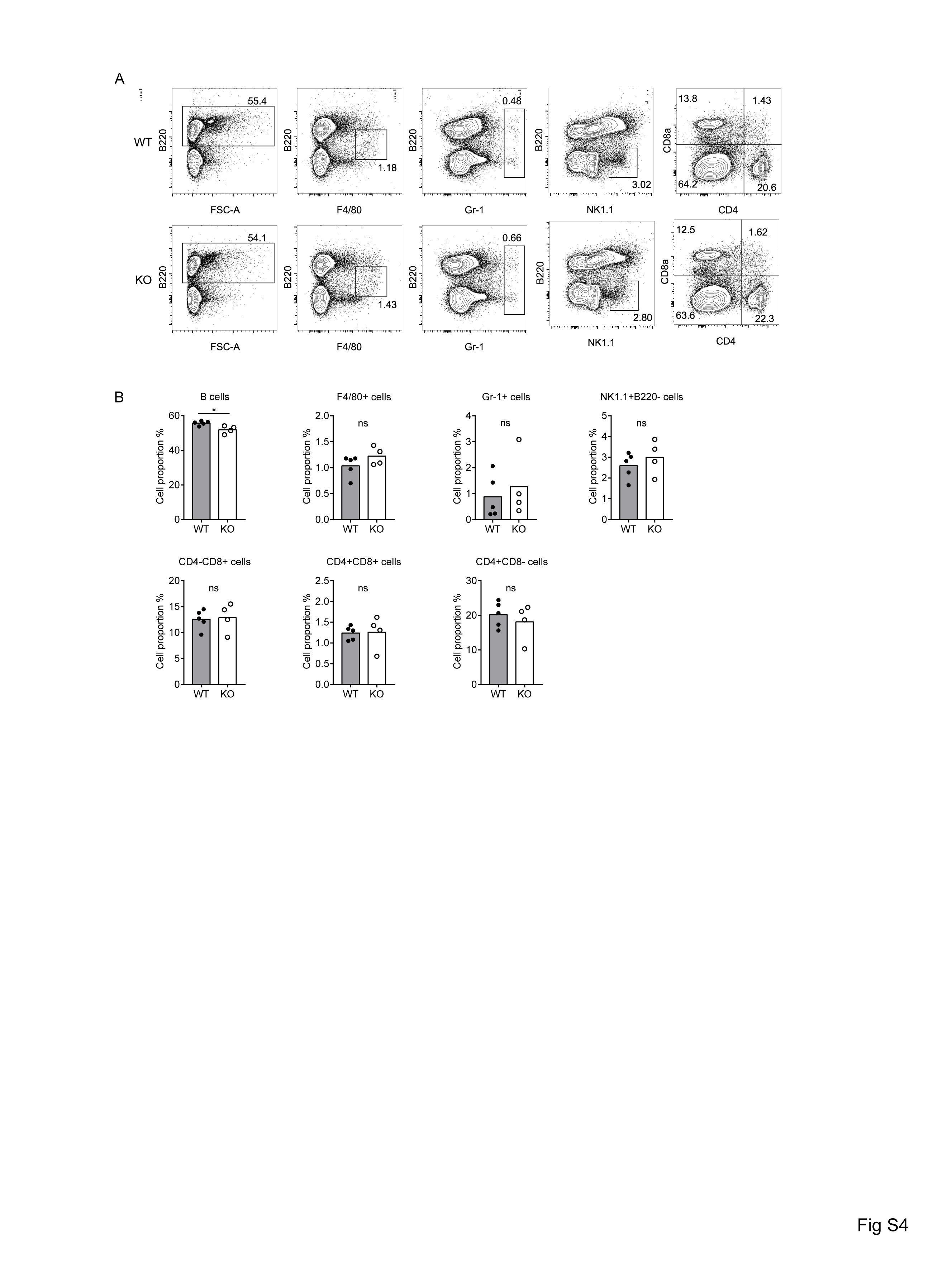

Supplement: Supplementary file 1 [file ijms-23-06584-s001.zip › Supplementary fig S4.tif]
